# The predictors for the non-compliance to follow-up among very low birth weight infants in the Korean neonatal network

**DOI:** 10.1371/journal.pone.0204421

**Published:** 2018-10-01

**Authors:** Nam Hyo Kim, Young Ah Youn, Su Jin Cho, Jong-Hee Hwang, Ee-Kyung Kim, Ellen Ai-Rhan Kim, Soon Min Lee, Korean Neonatal Network

**Affiliations:** 1 Department of Pediatrics, CHA Gangnam Medical Center, CHA University, Seoul, Korea; 2 Department of Pediatrics, Seoul St. Mary’s Hospital, College of Medicine, The Catholic University, Seoul, Korea; 3 Department of Pediatrics, Ewha Womans University, College of Medicine, Seoul, Korea; 4 Department of Pediatrics, Inje University College of Medicine, Ilsan Paik Hospital, Goyang, Korea; 5 Departmemt of Pediatrics, Seoul National University Children’s Hospital, Seoul, Korea; 6 Department of Pediatrics, Asan Medical Center Children’s Hospital, University of Ulsan College of Medicine, Seoul, Korea; 7 Department of Pediatrics, Gangnam Severance Hospital, Yonsei University College of Medicine, Seoul, Korea; Federal University of Sergipe, BRAZIL

## Abstract

The critical need to emphasize preterm infant follow-up after neonatal intensive care unit (NICU) discharge assures early identification of and intervention for neurodevelopmental disability. The aims of this study were to observe the follow-up rates in high-risk follow-up clinics, and analyze factors associated with non-compliance to follow-up among very low birth weight (VLBW) infants. The data was prospectively collected for 3063 VLBW infants between January 2013 and December 2014 from 57 Korean neonatal network (KNN) centers at a corrected age of 18–24 months. Correlations among demographic data, clinical variables, and neonatal intensive care unit (NICU) volume (divided into 4 quartiles) with the occurrence of non-compliance were analyzed. The overall follow-up rate at the corrected age of 18–24 month was 65.4%. The follow-up rates were inversely related to birth weight and gestational age. Apgar score, hospital stay, maternal age, and maternal education were significantly different between the compliance and non-compliance groups. The follow-up rate was higher for mothers with chorioamnionitis, abnormal amniotic fluid, multiple pregnancy, and *in vitro* fertilization. Infants with respiratory distress syndrome, bronchopulmonary dysplasia, patent ductus arteriosus ligation, periventricular leukomalacia, and retinopathy of prematurity were more common in the compliance group. Follow-up rates showed significant differences according to NICU volume. Using multivariate logistic regression, high birth weight, low NICU volume, siblings, foreign maternal nationality and high 5 min APGAR scores were significant independent factors associated with the non-compliance of VLBW infants for follow-up at 18–24 months of age. This is the first nation-wide analysis of follow-up for VLBW infants in Korea. Understanding factors associated with failure of compliance could help improve the long-term follow-up rates and neurodevelopmental outcomes through early intervention.

## Introduction

Improvement of survival rates for very low birth weight (VLBW) and extremely preterm infants resulted in advances in perinatal and neonatal care [1–3]. However, preterm infants are at a high risk for neurologic and developmental sequelae [4–6] and require significant outpatient services [7]. In Korea, over 3000 VLBW infants are born and discharged every year. The need to link high-risk infant tracking after discharge from the neonatal intensive care unit (NICU) has been emphasized to assure early identification and intervention [8]. Unfortunately, successful visits for outpatient services in recent studies are not guaranteed, with compliance rates between 70% to 100% with variations, even if neonates were identified as high-risk infants and careful follow-ups are required [9,10]. In a population-based study, there was also wide variability in referral among regions (8–98%) and NICUs (< 5–100%) [11].

High-risk infants who missed follow-up were more likely to present with severe impairment, such as cognitive impairment, or sensory adverse events. Prior research has found many factors to be associated with NICU follow-up appointment compliance [10,12–15]. To date, little is known about the risk factors associated with failure of referral to follow-up based on population-based analyses. From the California perinatal quality care collaboration [11], higher odds of high-risk infant follow-up (HRIF) referral were associated with lower birth weight, higher NICU volume, and California children's services regional level. Besides, lower odds were associated with small for gestational age and specific maternal race.

The Korean Neonatal Network (KNN) was established in 2013 to improve data collection systems in participating facilities and study various factors associated with mortality and morbidity of VLBW infants in Korea. Long-term data were also collected at the corrected age of 18–24 months and postnatal age of 3 years for VLBW infants. A better understanding of long-term outcomes in a national cohort will lead to improved approaches to neonatal care for VLBW infants in Korea. However, successful compliance with appropriate care and presentation to follow-up clinics must be guaranteed first and foremost.

The aims of this study were to observe the follow-up rates to high-risk follow-up clinics at the corrected age of 18–24 month, and to identify predictors of non-compliance to follow-up among VLBW infants in the KNN.

## Methods

### Study population

Data were prospectively collected for 3522 VLBW infants born between January 2013 and December 2014 from 57 KNN centers by local staff using a standardized electronic case report form [16,17]. The inclusion criteria for KNN was all VLBW infants born in or transferred to participating neonatal centers within 28 days of birth and surviving to discharge home. In this study, 459 preterm infants with deaths during the hospital stay were excluded. As a result, a total of 3063 infants were included.

This study was approved by the Samsung medical center institutional review board (2013-03-002). The data registry was approved by each institutional review boards of all 57 hospitals participating in the KNN. Written consent was obtained from the parents of infants during enrollment in KNN.

### Definition

Several factors potentially associated with non-compliance to follow-up clinics for VLBW infants were evaluated. Maternal, demographic, and NICU-related data were obtained through KNN records. Gestational age was determined by obstetric examination with ultrasonography early during the pregnancy or, via obstetric history based on the mother's last menstrual period. Birth weight was recorded for each baby as soon as they arrived at the NICU for admission. Bronchopulmonary dysplasia was defined by NIH classification [18]. Necrotizing enterocolitis (NEC) (≥ stage 2) was classified according to the system of Bell et al [19]. Severe intraventricular hemorrhage (IVH) was defined as more than graded II according to the method of Papile et al [20]. Mechanical ventilation was defined as the need for conventional or high frequency ventilation any time during the NICU stay. NICU volume was based on the total number of VLBW infants discharged between 2013 and 2014 for each NICU. NICU volume was then divided into 4 groups according to quartiles.

### Statistical analysis

Unadjusted comparisons of maternal demographics and neonatal characteristics between the compliant and non-compliant groups with high-risk follow-up were performed using a chi-squared or Fisher’s exact test for categorical data and Student’s t-test for continuous data. The correlations between compliance to follow-up clinics, gestational age, and birth weight were evaluated using Pearson’s correlation coefficients. Receiver operating characteristic (ROC) curve analysis with associated area under the curve (AUC) was conducted to explore the discriminate ability of NICU volume, gestational age, and birth weight in predicting compliance to follow-up clinics with the selection of the most suitable cut-off point of each parameter with the best sensitivity, specificity, and overall accuracy. Multivariate analyses of variables found to be significant on univariate analysis were also performed to identify independent predictors of non-compliance to follow-up clinics. All statistical analyses were performed using SPSS software version 25.0 (Armonk, NY: IBM Corp) and P values of < 0.05 were considered significant.

## Results

Among 3063 infants survived to discharge, 2003 infants (65.4%) were followed-up at the corrected age of 18–24 months. Significant differences were found for the following factors between the compliant and non-compliant groups, as shown in Table 1: mother with amniotic fluid volume abnormalities, multiple pregnancy, *in vitro* fertilization, maternal age, chorioamnionitis, maternal education level, maternal nationality, Apgar score, and siblings.

**Table 1:** Differences in relevant clinical variables between compliant and non-compliant groups.

	Compliant N = 2003	Non-compliant N = 1060	Total N = 3063	P Value
**Poly-and Oligo-hydramnios**	322 (17.2)	123 (13.0)	445 (15.8)	0.004
**Multiple pregnancies**	732 (36.5)	332 (31.3)	1064 (34.7)	0.004
**IVF**	455 (22.7)	197 (18.6)	652 (21.3)	0.008
**Siblings**	722 (36.0)	449 (42.4)	1171 (38.2)	0.001
**Maternal age, year**	32.83 [3.95]	32.32 [4.61]	32.66 [4.20]	0.002
**Maternal education level**				0.000
high school or less	426 (24.6)	263 (33.2)	689 (27.3)	
college or higher	1303 (75.4)	530 (66.8)	1833 (72.7)	
**Maternal nationality**				0.000
Korean	1954 (97.6)	1006 (94.9)	2960 (96.6)	
foreign country	49 (2.4)	54 (5.1)	103 (3.4)	
**GDM**	164 (8.2)	91 (8.6)	255 (8.3)	0.731
**PIH**	405 (20.2)	244 (23.0)	649 (21.2)	0.077
**Chorioamnionitis**	588 (34.1)	252 (29.8)	840 (32.7)	0.032
**PROM**	717 (36.0)	376 (35.8)	1093 (35.9)	0.937
**Male**	1013 (50.6)	524 (49.4)	1537 (50.2)	0.569
**C/sec**	1514 (75.6)	802 (75.7)	2316 (75.6)	1.000
**SGA**	446 (23.1)	255 (24.9)	701 (23.7)	0.296
**Gestational age, week**	29.02 [2.79]	26.65 [2.92]	29.23 [2.85]	0.000
**Birth weight, g**	1093.76 [270.31]	1158.56 [245.29]	1116.19 [263.69]	0.000
**Apgar score 1-minute**	4.73 [1.93]	5.03 [1.99]	4.84 [1.96]	0.000
**Apgar score 5-minute**	6.87 [1.65]	7.11 [1.69]	6.95 [1.67]	0.000
**Hospital day, d**	72.97 [36.90]	67.03 [39.22]	70.92 [37.82]	0.000

Data are expressed as number of patient (%) or mean [standard deviation]. IVF, *in vitro* fertilization; GDM, gestational diabetes mellitus; PIH, pregnancy-induced hypertension; PROM, premature rupture of membrane; C/Sec, caesarean section; SGA, small for gestational age; d, days; g, gram.

**Fig 1:**
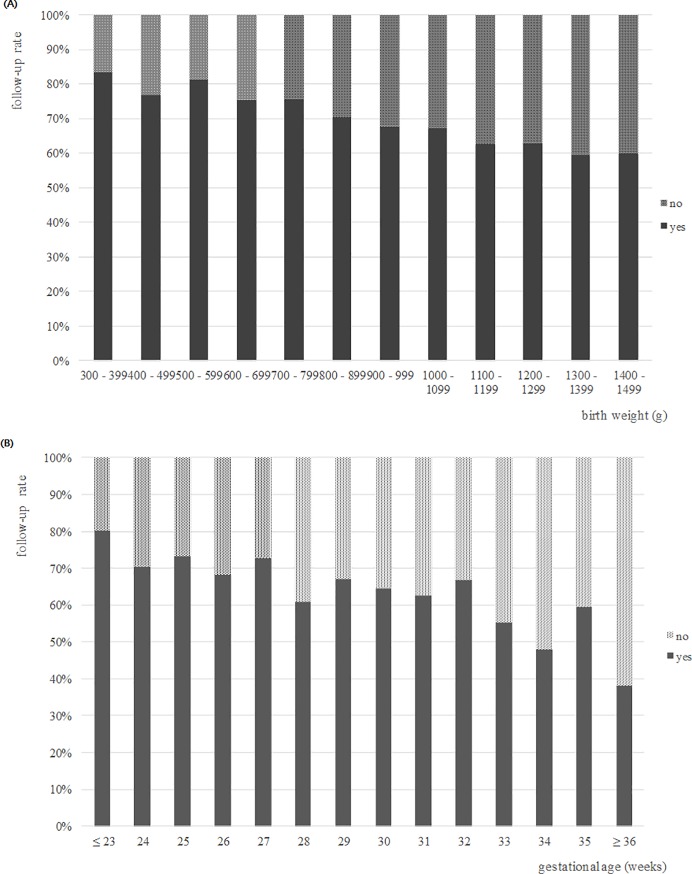
**Follow-up rates after NICU discharge of VLBW infants** by (A) birth weight and (B) gestational age.

Follow-up rates were significantly and inversely correlated with gestation and birth weight (P < 0.001) (Fig 1).

Neonatal morbidity such as respiratory distress syndrome (RDS), air leak, patent ductus arteriosus (PDA) ligation, periventricular leukomalacia (PVL), retinopathy of prematurity (ROP), and duration of supplement oxygenation showed significant differences between the compliant and not-compliant groups (Table 2).

**Table 2:** Comparisons of morbidities between compliant and non-compliant groups.

	Compliant N = 2003	Non-compliant N = 1060	P Value
**RDS**	1571 (78.4)	756 (71.3)	0.000
**Air leak**	82 (4.1)	25 (2.4)	0.013
**Pulmonary haemorrhage**	92 (4.6)	39 (3.7)	0.260
**Pulmonary hypertension**	96 (4.8)	46 (4.3)	0.589
**PDA medication**	739 (36.9)	362 (34.2)	0.133
**PDA ligation**	269 (12.9)	111 (10.5)	0.048
**BPD (≥ moderate)**	622 (31.2)	292 (27.9)	0.067
**IVH (≥ grade 2)**	279 (13.9)	132 (12.5)	0.266
**Post-haemorrhagic hydrocephalus**	68 (24.4)	39 (29.5)	0.280
**PVL**	154 (7.7)	105 (10.0)	0.034
**NEC (≥ stage 2)**	88 (4.4)	47 (4.4)	1.000
**Sepsis**	395 (19.7)	212 (20.0)	0.849
**ROP (≥ stage 2)**	482 (24.7)	201 (20.3)	0.007
**ROP operation**	213 (17.2)	73 (11.8)	0.002
**Abnormal hearing test**	296 (16.3)	108 (12.0)	0.003
**Congenital anomaly**	59 (2.9)	21 (2.0)	0.122
**Duration of invasive ventilator use, d**	16.08 [25.18]	14.18 [25.99]	0.049
**Duration of supplemental oxygen use, d**	9.51 [14.55]	8.27 [14.96]	0.028

Data are expressed as number of patient (%) or mean [standard deviation]. RDS, respiratory distress syndrome; PDA, patent ductus arteriosus; BPD, bronchopulmonary dysplasia; IVH, intraventricular haemorrhage; PVL, periventricular leukomalacia; NEC, necrotizing enterocolitis; ROP, retinopathy of prematurity; d, days.

NICU volume, as calculated according to the number of VLBW infants per institution enrolled in KNN, showed significant differences in follow-up rates. Higher NICU volume correlated with higher follow-up rates. The smallest NICU volume group, the 3^rd^ quartile, and the largest NICU volume group showed significant differences in the follow-up rate compared to others (Table 3).

**Table 3:** Comparative analysis of compliance to follow-up clinic after discharge according to NICU volume.

	**Compliant** **N = 2003**	**Non-compliant** **N = 1060**	**P Value**
**NICU volume**^**+**^			0.000
Group 1	436 (56.0)	343 (44.0)	
Group 2	505 (64.1)	283 (35.9)	
Group 3	495 (57.7)	363 (42.3)	
Group 4	567 (88.9)	71 (11.1)	
	**OR**	**95% CI**	**P Value**
** Group 1 vs. 2,3,4**	0.582	0.492, 0.687	0.000
** Group 2 vs. 1,3,4**	0.926	0.781, 1.096	0.371
** Group 3 vs. 1,2,4**	0.630	0.536, 0.741	0.000
** Group 4 vs. 1,2,3**	5.500	4.242, 7.131	0.000

Data are expressed as number of patient (%).

^**+**^ Quartile for NICU volume is based on average annual VLBW discharge volume for 2013 and 2014, expressed as mean ± standard deviation. 1^st^ quartile, 26.4 ± 14.1; 2^nd^ quartile, 70.5 ± 11.3; 3^rd^ quartile, 141.6 ± 32.6; 4^th^ quartile, 239.7 ± 23.6.

A ROC curve analysis was conducted to explore factors predicting non-compliance to follow-up clinic. ROC analysis for birth weight and follow-up (P < 0.001) showed an AUC of 0.567 (sensitivity of 55.9% and specificity of 54.4% for a cutoff of 1162.5 g). For gestational age and follow-up (P < 0.001), the AUC was 0.560, with a sensitivity of 54.2% and specificity of 53.5% for a cutoff of 29.2 weeks. For NICU volume and follow-up (P < 0.001), the AUC was 0.612, with a sensitivity 53.0% and specificity of 59.1% for a cutoff of 3^rd^ quartile of NICU volume.

In multiple logistic regression, foreign maternal nationality, siblings, 5-min APGAR score, birth weight, and NICU volume were confirmed as significant predictors of non-compliance to follow-up (Table 4).

**Table 4:** Results of multivariable logistic regression model for non-compliance to follow-up clinic for VLBW infants.

	OR	95% CI	P Value
**IVF**	0.869	0.642, 1.178	0.366
**Siblings**	1.311	1.028, 1.673	0.029
**Maternal age**	0.972	0.945, 1.000	0.047
**Maternal education level**			
high school or less	1.231	0.962, 1.575	0.099
college or higher	1.0	reference	
**Maternal nationality**			
Korean	1.0	reference	
foreign country	2.059	1.090, 3.889	0.026
**Poly-and Oligo-hydramnios**	1.114	0.813, 1.526	0.501
**Chorioamnionitis**	0.944	0.744, 1.198	0.634
**Air leak**	1.497	0.718, 3.123	0.282
**RDS**	1.111	0.829, 1.490	0.480
**Apgar score 5-minute**	1.123	1.040, 1.213	0.003
**PDA ligation**	0.762	0.517, 1.124	0.171
**ROP (≥ stage 2)**	1.052	0.753, 1.471	0.765
**PVL**	0.759	0.477, 1.209	0.246
**Abnormal hearing test**	1.141	0.831, 1.567	0.414
**Duration of supplemental oxygen use**	0.992	0.983, 1.001	0.074
**Hospital day**	1.003	0.998, 1.008	0.261
**NICU volume**			0.000
Group 1	5.422	3.696, 7.953	0.000
Group 2	3.652	2.484, 5.370	0.000
Group 3	5.877	4.080, 8.467	0.000
Group 4	1.0	reference	
**Birth weight**			0.028
≤ 750 g	1.0	reference	
751–1000 g	1.324	0.829, 2.115	0.239
1001–1250 g	1.513	0.908, 2.523	0.112
1251–1499 g	2.044	1.193, 3.504	0.009

VLBW, very low birth weight; IVF, *in vitro* fertilization; RDS, respiratory distress syndrome; PDA, patent ductus arteriosus; ROP, retinopathy of prematurity; PVL, periventricular leukomalacia; NICU, neonatal intensive care unit

## Discussion

Preterm infant follow-up clinics after NICU discharge play important roles in monitoring growth and development; provides continuity of medical care; and provides assurance of appropriate therapeutic interventions [21]. High follow-up rates have positive impacts on post-discharge health care and also allows NICU treatment to be assessed after discharge, as well saving time and money [9,10,22]. This is the first study to analyze the follow-up rates and factors associates with non-compliance to follow-up as a nation-wide study in Korea. Understanding factors associated with non-compliance to follow-up is a starting point for providing targeted intervention [9].

Lack of developmental follow-up has been associated with higher rates of neurodevelopmental impairment. The American Academy of Pediatricians recommends that preterm infants be followed-up by physicians affiliated with a NICU [21,23]. However, although established NICU follow-up clinics exist, successful follow-up is not assured, and follow-up rates are highly variable. Recent studies have reported compliance rates ranging from below 70% up to 100%, so quality improvement initiatives aim to increase these rates. Variations depend on the local situations, with inter-hospital and international variability noted.

Prior research has identified many factors associated with NICU follow-up appointment compliance. Maternal drug use (odds ratio [OR] = 0.049), multiple gestation pregnancy (OR = 0.163), male sex (OR = 0.308), and greater distance from the hospital (OR = 0.987) [9] were independently associated with reduced appointment compliance. Others showed multiple gestations, postnatal glucocorticoids, chronic lung disease, and multiple morbidities have been associated with compliance [12,14,24–28]. In this study, siblings, birth weight, NICU volume, maternal foreign nationality, and 5-min APGAR score were confirmed as significant independent predictors of increasing non-compliance to follow-up.

In this study, the compliant group had higher rates of multiple pregnancies. Previous reports showed that non-compliance after a multiple pregnancy might be due to the fact that caring for two sick newborns puts even more stress on the resources and time of a family than caring for one newborn [9]. This difference can explain why IVF is more common in VLBW infants in Korea [29,30] than in the USA in 2014 [31], and multiple pregnancy rates showed higher trends in Korea than other countries. These are highly correlated because there is an increasing likelihood that an artificial pregnancy will conceive the twins [29]. The parents may have much concern for neurodevelopment disability in preterm twin babies conceived through IVF in Korea. In accordance with a previous study, non-compliance to follow-up in cases involving multiple children may be explained by the shortage of time and resources of parents unlike cases involving only one child. It has been reported that male infants are associated with greater non-compliance than females [12], however, we identified no sexual difference in terms of follow-up rates. Higher rates of compliance among infants with longer NICU stays and more days on oxygen remained independently significant, consistent with our study [9]. However, they were not identified as independent predictors for the non-compliance to follow-up on multivariate analysis. Children of older mothers were more likely to attend follow-up (30 years vs 27 years) [28]. In this study, the compliant group showed a significantly older maternal age.

Higher NICU volume was associated with greater odds for high-risk infant follow-up referrals [11]. In this study, NICU volume was significantly associated with compliance to follow-up in clinics. The NICU with the highest quartile NICU volume showed the five times higher compliance with follow-up compared with the lowest quartile NICU volume. Similar to studies demonstrating the differences in personnel, resources, and approaches among follow-up clinics, we identified broad variations in the availability, process, and approach to follow-up clinics [22,32,33]. More financial and policy support to improve the follow-up rate from lower volume NICU centers is needed.

The ranked order of variables for predicting compliance to follow-up, as assessed by AUC analysis is as follows: NICU volume (0.612), birth weight (0.567), and gestational age (0.560) with poor prediction. It may be caused by the fact that compliance to follow-up is related with more than a single birth variable. Multiple variables, such as comorbidities and maternal factors, may exist. NICU volume ≥ 3^rd^ quartile, gestational age ≤ 29 weeks, and birth weight ≤ 1160 g were found to be significantly associated with compliance to follow-up. The parents of infants with a large gestational age and a high birth weight reflects the feeling of decreased need for follow-up.

In order to improve the follow-up rate, it is essential to make an appropriate follow-up appointment at the time of NICU discharge [9]. However, there has been much variation concerning when and how to make follow up clinic appointments. After establishing the follow-up registry in KNN, all infants born at VLBW are expected to have scheduled appointments at a follow-up clinic at the corrected age of 18–24 month and at postnatal 3 years of age. A regular announcement system in the KNN concerning the need for infants to visit follow-up clinics could be created to remind physicians to encourage referral to follow-up clinics. Furthermore, annual education concerning the importance of follow-up clinic appointments, real-time display for the follow-up rate of each hospital, and regular reporting on the follow-up rate with comparisons to other hospitals would allow for improved consistency in follow-up clinics.

Various interventions could potentially be used to increase the follow-up rate after NICU discharge. To provide patient education through printouts, conversations with primary health care physicians, and videos can help families recognize the importance of follow-up clinics. Reminder letters and phone calls have also been used with success [34]. In particular, we suggest it is necessary to target the infants with factors including mother with foreign nationality, infants with siblings, high APGAR score, and higher birth weight for intervention.

The limitation of this study was that it narrowly defined compliance to follow-up as collecting the data at the corrected age of 18–24 months. Families ultimately attending a follow-up clinic during a different age window were considered non-compliant. Furthermore, the non-compliant group may also include cases of death after discharge. NICU volume assumed as total number of annual enrollments in KNN during study period, it may have variation within the year and sites. Additionally, regional aspects such as distance, and resources for the hospital were not analyzed.

In conclusion, through this first nationwide study, we detected the factors associated with non-compliance to follow-up at the corrected age of 18–24 month. Strategies, such as patient education, reminder phone calls, and an organized system of storing and remembering appointments, should be used particularly targeted to families who may be at risk of non-compliant to follow-up based on the factors we have identified. This could contribute to improve patient care accordingly and long-term outcome through early detection and intervention.
